# Comparative Analysis of Flesh Quality in Triploid and Allotetraploid Pengze Crucian Carp: Nutritional Composition, Flavor Profile, Texture Properties, and Metabolomics Insights

**DOI:** 10.3390/biology15050429

**Published:** 2026-03-05

**Authors:** Gang He, Menglu Li, Wen Xie, Jiaxin Yuan, Yonghui Deng, Yali Yu, Jiawei Wang, Zhiying Tao, Huiming Zhou, Liyun Ding, Jun Xiao, Yongyao Yu, Zexia Gao, Weimin Wang, Hong Liu

**Affiliations:** 1 College of Fisheries, Huazhong Agricultural University, Wuhan 430070, China; 2 Jiangxi Fisheries Research Institute, Nanchang 330039, China; 3 Yangtze River Fisheries Research Institute, Chinese Academy of Fishery Sciences Ministry, Wuhan 430223, China

**Keywords:** Pengze crucian carp, triploid, allotetraploid, flesh quality, metabolomics

## Abstract

This study provides a systematic and multidimensional evaluation of flesh quality differences between triploid (T-PZ) and allotetraploid (A-PZ) Pengze crucian carp, addressing a significant gap in genetic improvement of this economically important aquaculture species. By integrating proximate composition analysis, detailed amino acid and fatty acid profiling, volatile flavor compounds, texture characteristics, and non-targeted muscle metabolomics, we delineated the distinct quality attributes associated with each cytotype. The results showed that T-PZ possessed significantly higher crude protein and histidine content, along with a superior flavor profile characterized by lower relative levels of fishy odor-associated aldehydes such as hexanal, heptanal, and nonanal. In contrast, A-PZ exhibited significantly elevated crude lipid, total fatty acid, SFA, MUFA, PUFA, and EPA + DHA contents; a higher essential amino acid index; and improved tenderness indicated by significantly lower hardness and chewiness. Metabolomic analysis identified 216 significantly different metabolites, notably enriched in key pathways including glycine, serine, and threonine metabolism; arginine biosynthesis; and glycerophospholipid metabolism. These comprehensive findings elucidate the complementary nutritional and sensory strengths of the two ploidy forms, thereby establishing a crucial scientific foundation for targeted, quality-driven breeding programs aimed at optimizing flesh quality in Pengze crucian carp for the aquaculture industry.

## Simple Summary

This study compared the flesh quality of triploid and allotetraploid Pengze crucian carp to understand how ploidy affects nutritional and sensory traits. We analyzed their muscle composition, amino acid and fatty acid profiles, flavor compounds, texture, and metabolomics. Results showed that triploids had higher protein content and a fresher flavor with fewer fishy odor compounds. Allotetraploids contained more healthy polyunsaturated fatty acids (including EPA and DHA), a better amino acid balance, and were more tender. Metabolomics revealed 216 different metabolites between the two groups, linked to key pathways like amino acid and lipid metabolism. These findings provide a scientific basis for breeding programs aimed at improving fish quality for consumers.

## 1. Introduction

Pengze crucian carp (*Carassius auratus* var. *Pengze*), officially recognized as China’s first superior aquaculture variety directly selected from wild crucian carp, has become a cornerstone of freshwater aquaculture due to its desirable traits such as rapid growth, hypoxia tolerance, strong disease resistance, and high nutritional value [1]. Since its approval in 1996 (GS-01-003-1996), it has supported a large-scale industry with significant economic benefits. The nutritional appeal of fish, particularly its high-quality protein, beneficial lipids, and micronutrients, has driven increasing global consumption, placing greater emphasis on the intrinsic quality attributes of farmed species. In this context, flesh quality—encompassing nutritional composition, texture, flavor, and visual appearance—has emerged as a critical determinant of consumer acceptance, market price, and ultimately, the sustainability and profitability of aquaculture operations.

However, the sustainable production of Pengze crucian carp is increasingly threatened by challenges, including inbreeding depression, germplasm degradation, and declining stress resistance, which can negatively impact both yield and quality traits [2]. These issues highlight an urgent need for genetic improvement and the development of enhanced varieties that combine high productivity with superior flesh quality. Genetic breeding strategies, such as ploidy manipulation and hybridization, offer powerful tools to achieve these goals. Polyploidy, in particular, can induce significant phenotypic and physiological changes, often leading to altered growth performance, environmental adaptability, and product quality in aquatic species.

Gynogenetic breeding using heterologous sperm has emerged as a particularly viable strategy for the genetic enhancement of Pengze crucian carp [3,4]. Studies have consistently shown that offspring induced by sperm from sea carp (*Cyprinus acutidorsalis*) and Heilongjiang carp (*C. carpio haematopterus*) exhibit significant growth advantages and improved morphological traits, validating the potential of this method [5,6,7]. A pivotal advancement was the subsequent discovery of allotetraploid individuals in the gynogenetic progeny of Pengze crucian carp induced with sperm from Xingguo red carp [8]. These individuals were confirmed through karyotype analysis (4n = 200+) to be products of distant hybridization. Compared to the conventional triploid form, the novel allotetraploid line demonstrates superior performance in several key zootechnical areas, including enhanced growth rates, shorter embryonic development time, and stronger resistance to crucian carp herpesvirus, making it a promising candidate for future breeding applications [9,10,11,12,13].

Despite these well-documented advantages in growth, development, and disease resistance, a comprehensive and systematic assessment of its flesh quality—encompassing nutritional value, sensory attributes, and textural properties—remains conspicuously absent. Flesh quality is a complex, multi-parameter trait directly influencing consumer preference and commercial success [14,15]. While metabolic profiles underlie many quality attributes, the specific molecular mechanisms differentiating triploid and allotetraploid muscle metabolism are unknown. This critical knowledge gap limits the ability of breeders to make informed, quality-oriented selection decisions and to fully exploit the commercial potential of the novel allotetraploid line.

Therefore, this study conducts an integrated comparative analysis of key flesh quality indicators—including proximate composition, amino acid and fatty acid profiles, volatile flavor compounds, texture parameters, and muscle metabolomics—between stable triploid and fourth-generation allotetraploid Pengze crucian carp. The application of metabolomics is particularly aimed at uncovering the biochemical pathways responsible for the observed phenotypic differences in quality. Overall, this work aims to fill a critical research gap, provide a robust scientific basis for the quality-focused genetic breeding of Pengze crucian carp, and ultimately support the strategic development and promotion of high-quality, market-competitive varieties for a sustainable aquaculture future.

## 2. Materials and Methods

### 2.1. Fish and Feeding Management

The triploid fish were sourced from the Jiangxi Pengze Crucian Carp Breeding Farm, and the allotetraploid fish were sourced from Huazhong Agricultural University. After a 4-week rearing period in containers (6 m × 2 m × 2 m) at the Jiangxi Fisheries Research Institute base, 2400 healthy fish of uniform size were randomly selected and divided into the T-PZ group (initial weight 80.55 ± 5.08) and A-PZ group (initial weight 84.83 ± 4.79 g), with 3 containers per group (800 fish per container). The fish were fed a high-quality commercial crucian carp diet containing 32% crude protein and 4% crude lipid (Jiangxi Wujiahao Agricultural and Animal Husbandry Trade Co., Ltd., Nanchang, China). The fish were fed twice daily at 9:00 and 17:00, with feeding amounts adjusted based on fish intake, water temperature, and weather conditions. During the experiment, water quality was regularly monitored, maintaining dissolved oxygen ≥ 5.6 mg/L, ammonia nitrogen ≤ 0.2 mg/L, nitrite ≤ 0.1 mg/L, pH 7.8–8.5, and water temperature 22–30 °C. The farming trial lasted 8 weeks.

### 2.2. Sample Collection

**Figure 1:**
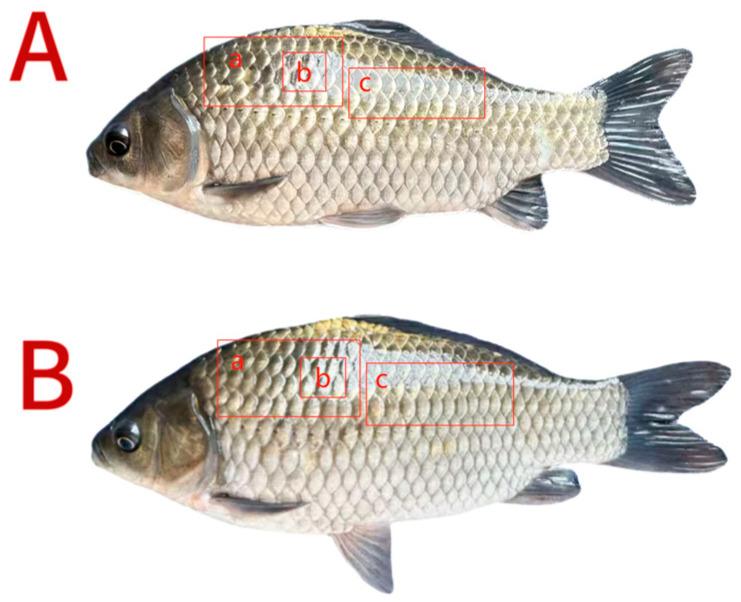
The sampling sites for flesh quality analyses. (**A**) T-PZ; (**B**) A-PZ. a: Color parameters; b: texture characteristics; c: proximate composition, amino acids, fatty acids and volatile flavor components.

After the feeding trial, the fish were starved for 24 h before sampling, then counted and weighed per individual container to calculate survival, weight gain (WG) and feed conversion ratio (FCR). From each parallel, three fish were randomly selected, and blood was collected from the caudal vein of each fish. Subsequently, dorsal muscle from above the lateral line was collected, and the color of the surface was measured. Subsequently, two blocks of dorsal muscle (0.5 cm^3^ each) were sampled from the left and right sides for immediate texture analysis. The remaining muscle tissue was homogenized and stored at −80 °C for determining flesh proximate composition, amino acid and fatty acid composition and volatile flavor compounds. Additionally, two fish were selected from each parallel. The white dorsal muscle above the lateral line was collected into enzyme-free tubes, immediately flash-frozen in liquid nitrogen, and subsequently transferred to −80 °C for metabolomics analysis. The sampling sites for muscle quality analysis are illustrated in Figure 1.

### 2.3. Measurement Indicators and Methods

#### 2.3.1. Measurement of Growth Performance

Survival, weight gain (WG), feed conversion ratio (FCR), and condition factor (CF) were calculated as follows:

Survival (%) = 100 × (final fish number/initial fish number).

WG (%) = 100 × [(final weight (g) − initial weight (g))/initial weight (g)].

FCR = feed intake (dry weight, g)/[final weight (g) − initial weight (g)].

CF (g/cm^3^) = 100 × [final body weight (g)/body length (cm)^3^].

#### 2.3.2. Determination of Proximate Composition in Flesh

The moisture, ash, crude lipid and crude protein contents in the muscle were analyzed following the method of AOAC [16]. Moisture content was measured by drying samples to constant weight at 105 °C in a drying oven. Then the dried samples were ground into powder for further determination. Lipid content was measured following the Soxhlet extraction method. Protein content was measured using the Kjeldahl method. Ash content was measured by combusting samples in a muffle furnace at 550 °C for 6 h. All nutritional component contents were calculated based on fresh weight.

#### 2.3.3. Determination of Amino Acids

To determine the amino acid composition, samples of flesh (0.02 g) were placed in hydrolysis tubes with 10 mL of 6 mol/L hydrochloric acid, vacuum-sealed, and hydrolyzed at 110 °C for 22 h. After cooling, the solution was diluted to 25 mL with pure water. A 5 mL aliquot was then further diluted to 10 mL, and 5 mL of this diluted solution was transferred to a small weighing bottle. The sample was evaporated to dryness in an 80 °C water bath. Subsequently, 1 mL of deionized water was added and evaporated to dryness, with this process repeated three times. The residue in the weighing bottle was dissolved in 5 mL of 0.02 mol/L hydrochloric acid, transferred to a centrifuge tube, and thoroughly mixed. A 1 mL portion of the solution was filtered through a 0.22 μm aqueous-phase membrane into an injection vial and analyzed using an L-8900 amino acid analyzer (Hitachi, Tokyo, Japan).

The nutritional value of amino acids was evaluated based on the ideal protein essential amino acid scoring pattern recommended by the FAO/WHO and the whole egg protein amino acid pattern. Amino acid score (AAS), chemical score (CS), and essential amino acid index (EAAI) were calculated as follows:

aa = (flesh amino acid content/Muscle protein content) × 6.25 × 1000

AAS = aa/AA_(FAO/WHO)_

CS = aa/AA_Egg_

EAAI = [(100A/AE) × (100B/BE) × … × (100I/IE)]^1/n^

In the formula, aa is the content of certain amino acids in the sample (mg/g N); AA (FAO/WHO) represents the content of the same amino acids in the FAO/WHO standard scoring mode (mg/g N); AA_Egg_ represents the content of the same amino acids in the whole egg protein (mg/g N); n is the number of essential amino acids for comparison; A, B, …, I are the content of each essential amino acid in the sample (mg/g N); and AE, BE, …, IE is the content of the essential amino acids corresponding to the whole egg protein (mg/g N).

#### 2.3.4. Determination of Fatty Acids

To determine the fatty acid composition, a flesh sample (1.00 g) was weighed into a hydrolysis tube, mixed with 1 mL of 10,000 mg/L glyceryl tricaprate (internal standard), and hydrolyzed with 10 mL hydrochloric acid. The hydrolysate was extracted using 25 mL of an ether + petroleum ether (1:1) mixture, and the extract was concentrated to dryness via rotary evaporation. Subsequently, 8 mL of 2% sodium hydroxide-methanol solution was added for saponification, followed by 7 mL of 14% boron trifluoride-methanol solution for methylation. Finally, 10 mL of n-hexane was added, and the mixture was vortexed and allowed to stratify. The supernatant (1 mL) was collected into a sample vial for analysis using an Agilent 7890A gas chromatograph (Agilent Technologies, Santa Clara, CA, USA).

#### 2.3.5. Flesh Texture Characteristics and Color Parameters

According to the method of Sun et al. [17], the flesh samples of Pengze crucian carp were cut into small pieces of 0.5 cm^3^, and the texture characteristics of the samples were tested by using a texture analyzer (model TVT-300XP, Sweden Taiwo Company, Goteborg, Sweden). The test conditions: the probe was a P-cy5s cylindrical probe, the velocity before and after the test was 5 mm/s, the test velocity was 1 mm/s, the compression ratio was 30%, the trigger force was 10 g, and the time interval between two compressions was 5 s.

Color was measured using a Minolta colorimeter (CR-400, Konica Minolta Co., Ltd., Osaka, Japan) at three different parts of the dorsal muscle, including L* (lightness; higher values indicate greater brightness), a* (redness; positive values denote red intensity), and b* (yellowness; positive values represent yellow intensity).

#### 2.3.6. Determination and Evaluation of Volatile Flavor Compounds

Referring to the method of Lv et al. [18], 2.0 g of muscle tissue was placed into a headspace vial, mixed with 8 mL of saturated NaCl solution and 1 µL of 1000 mg/L cyclohexanone. A DVB/CAR/PDMS (50/30 μm) extraction fiber was inserted for headspace solid-phase microextraction (HS-SPME) at 70 °C for 30 min. The extracted volatile compounds were analyzed using a Thermo TSQ 9000 triple quadrupole GC-MS system (Thermo Fisher Scientific, Singapore).

Determination of key flavor compounds: The Relative Odor Activity Value (ROAV) method was applied to determine the key flavor compounds. The component with the greatest contribution to the overall flavor profile was assigned ROAV_max_ = 100. For other volatile compounds, ROAV was calculated using the following formula:


$$ROVA\approx \frac{C_{A}}{C_{\mathrm{stan}}}\times \frac{T_{\mathrm{stan}}}{T_{A}}\times 100$$

In the formula, C_A_ = relative content of the volatile compound (μg/kg); T_A_ = odor threshold of the volatile compound (μg/kg); C_stan_ = relative content of the most dominant flavor compound (μg/kg); and T_stan_ = odor threshold of the most dominant flavor compound (μg/kg).

#### 2.3.7. Analysis of Flesh Metabolomics

To 100 mg of flesh, 1 ml of cold 90% methanol was added. The lysate was homogenized by MP homogenizer (MP Biomedicals, Solon, OH, USA) (24 × 2, 6.0 M/S, 60 s, twice). The homogenate was sonicated at low temperature (30 min/once, twice). The mixture was centrifuged for 20 min (14000× *g*, 4 °C). The supernatant was dried in a vacuum centrifuge. For LC-MS analysis, the samples were re-dissolved in 100 μL acetonitrile/water (1:1, *v*/*v*) solvent.

Analysis was performed using a UHPLC (1290 Infinity LC, Agilent Technologies, Santa Clara, CA, USA) coupled to a quadrupole time-of-flight (AB Sciex TripleTOF 6600, SCIEX, Framingham, MA, USA). For HILIC separation, samples were analyzed using a 2.1 mm × 100 mm ACQUIY UPLC BEH 1.7 µm column (Waters, Co. Wexford, Ireland). In both ESI positive and negative modes, the mobile phase contained A = 25 mM ammonium acetate and 25 mM ammonium hydroxide in water and B = acetonitrile. The gradient was 85% B for 1 min and was linearly reduced to 65% in 11 min, and then was reduced to 40% in 0.1 min and kept for 4 min, and then increased to 85% in 0.1 min, with a 5 min re-equilibration period employed. The ESI source conditions were set as follows: Ion Source Gas1 as 60, Ion Source Gas2 (Gas2) as 60, curtain gas (CUR) as 30, source temperature—600 °C, IonSpray Voltage Floating (ISVF) ±5500 V. In MS-only acquisition, the instrument was set to acquire over the m/z range 60–1000 Da, and the accumulation time for TOF MS scan was set at 0.20 s/spectra. In auto MS/MS acquisition, the instrument was set to acquire over the m/z range 25–1000 Da, and the accumulation time for product ion scan was set at 0.05 s/spectra. The product ion scan is acquired using information-dependent acquisition (IDA) with high sensitivity mode selected. The parameters were set as follows: the collision energy (CE) was fixed at 35 V with ± 15 eV; declustering potential (DP), 60 V (+) and −60 V (−); excluding isotopes within 4 Da, candidate ions to monitor per cycle—10. All samples were analyzed in a randomized order to avoid batch effects. Pooled QC samples were injected periodically throughout the run to monitor system stability and condition the column. Data normalization was performed using probabilistic quotient normalization (PQN) based on the QC samples to correct for signal drift and ensure comparability.

The raw MS data were converted to MzXML files using ProteoWizard MSConvert (v3.0.6428) before importing into freely available XCMS software (online 3.7.1). For peak picking, the following parameters were used: centWave m/z = 10 ppm, peakwidth = c (10, 60), prefilter = c (10, 100). For peak grouping, bw = 5, mzwid = 0.025, minfrac = 0.5 were used. CAMERA (Collection of Algorithms of MEtabolite pRofile Annotation) was used for the annotation of isotopes and adducts. In the extracted ion features, only the variables having more than 50% of the nonzero measurement values in at least one group were kept. Compound identification of metabolites was performed by comparing the accuracy m/z value (<10 ppm) and MS/MS spectra with an in-house database established with available authentic standards. The missing data were filled by the KNN (K-Nearest Neighbor) method, and features with RSD greater than 50% are filtered out.

### 2.4. Statistical Analysis

The experimental data were presented as mean ± standard deviation. All data were analyzed by SPSS 26.0 One-way analysis of variance (one-way ANOVA), with *p* < 0.05 denoting a significant difference and *p* < 0.01 indicating an extremely significant difference. Tukey’s multiple range tests were used to determine the statistical significance between groups.

## 3. Results

### 3.1. Growth Performance

As shown in Table 1, the final body weight (FBW), weight gain (WG) and survival of the T-PZ group were significantly lower than those of the A-PZ group (*p* < 0.05). No significant differences were observed in feed conversion ratio (FCR) and condition factor (CF) between the T-PZ and A-PZ groups (*p* > 0.05).

### 3.2. Flesh Nutrient Composition

As shown in Table 2, the crude lipid content of the A-PZ group (3.38 g/100 g) was significantly higher than that of the T-PZ group (2.70 g/100 g) (*p* < 0.01). In contrast, the crude protein content of the A-PZ group (19.20 g/100 g) was extremely lower than that of the T-PZ group (19.77 g/100 g) (*p* < 0.01). No significant differences were observed in the flesh moisture and crude ash content between the T-PZ and A-PZ groups (*p* > 0.05).

### 3.3. Flesh Amino Acid Composition and Nutritional Value

In Table 3, the total amino acid (TAA) and total essential amino acid (EAA) contents of T-PZ were slightly higher than those of A-PZ (*p* > 0.05), with increases of 0.98% and 1.46%, respectively. The histidine content in the flesh of the T-PZ group was significantly higher than that of the A-PZ group (*p* < 0.01), whereas no significant differences were observed in the contents of other amino acids between the two groups (*p* > 0.05). In both groups, glutamic acid was the most abundant amino acid, and cysteine was the least abundant in the flesh. The ∑EAA/∑TAA ratio exceeded 40% in both groups.

As shown in Table 4, the total EAA content, as well as the levels of threonine, isoleucine, and phenylalanine + tyrosine in the flesh of Pengze crucian carp from both groups, were higher than those in the FAO/WHO amino acid pattern but lower than those in the whole egg protein amino acid model. In contrast, the contents of leucine and lysine were higher than those in the whole egg protein standard, with the highest levels observed in A-PZ, being 1.20- and 1.13-fold higher than the whole egg protein standard, respectively.

As shown in Table 5, under both the amino acid score (AAS) and chemical score (CS) standards, methionine + cysteine and valine were identified as the first and second limiting amino acids in both T-PZ and A-PZ, respectively. The highest scores were lysine and leucine, respectively. The EAA index (EAAI) of T-PZ was 82.17, slightly lower than that of A-PZ (84.28).

### 3.4. Flesh Fatty Acid Composition

As shown in Table 6, sixteen fatty acids were detected in the flesh of both groups, including four saturated fatty acids (SFAs), four monounsaturated fatty acids (MUFAs), and eight polyunsaturated fatty acids (PUFAs). In both groups, oleic acid (C18:1n9c) was the most abundant fatty acid (779.01 mg/100 g and 1010.31 mg/100 g, respectively), followed by linoleic acid (C18:2n6c) (509.62 mg/100 g and 661.11 mg/100 g, respectively). Compared with T-PZ, the contents of γ-linolenic acid and arachidonic acid were significantly lower in A-PZ (*p* < 0.01), whereas the contents of the other fatty acids were increased. The totals for fatty acids (∑FA), saturated fatty acids (∑SFA), monounsaturated fatty acids (∑MUFA), polyunsaturated fatty acids (∑PUFA), and EPA + DHA contents in A-PZ were significantly higher than those in T-PZ (*p* < 0.01). However, the ∑n − 3 PUFA/∑n − 6 PUFA ratio was not significantly different between the two groups (*p* > 0.05).

### 3.5. Flesh Texture and Color Parameters

**Figure 2:**
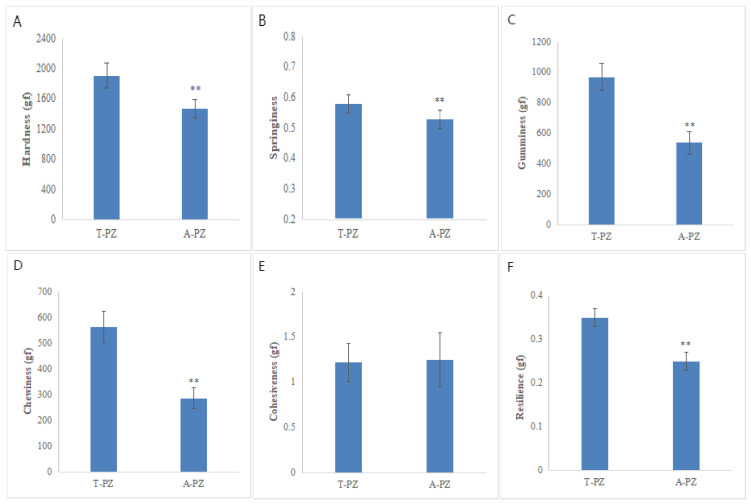
Comparison of flesh texture characteristics including hardness (**A**), springiness (**B**), gumminess (**C**), chewiness (**D**), cohesiveness (**E**), and resilience (**F**) between the T-PZ and A-PZ groups (*n* = 6). ** indicates significant difference (*p* < 0.05).

As shown in Figure 2 and Table 7, significant differences in flesh texture characteristics were observed between the T-PZ and A-PZ groups. Compared with T-PZ, flesh hardness, springiness, gumminess, chewiness, and resilience were significantly lower in A-PZ (*p* < 0.05). In contrast, there were no significant differences in cohesiveness and in the color parameters L*, a* and b* between the two groups (*p* > 0.05).

### 3.6. Flesh Volatile Flavor Compounds

As shown in Table 8, a total of 55 volatile compounds were detected in the flesh. Among these, 53 compounds were identified in the T-PZ group, including 18 aldehydes, 3 ketones, 6 alcohols, 16 esters, and 10 other compounds, whereas 51 compounds were detected in the A-PZ group, comprising 17 aldehydes, 3 ketones, 5 alcohols, 15 esters, and 11 other compounds. Among the detected compounds, hexanal, nonanal, 2,5-octanedione, 1-hexanol and 1-octen-3-ol were present at relatively high levels in both groups. In addition, 4-pentylbenzaldehyde, 1-nonanol and butanedioic acid bis(2-methylpropyl) ester were detected only in T-PZ, whereas 2,2,4-trimethyl-1,3-pentanediol diisobutyrate and butylated hydroxytoluene were detected exclusively in A-PZ. The contents of aldehydes, ketones, alcohols, esters and other compounds in T-PZ were significantly lower than those in A-PT (*p* < 0.01).

**Figure 3:**
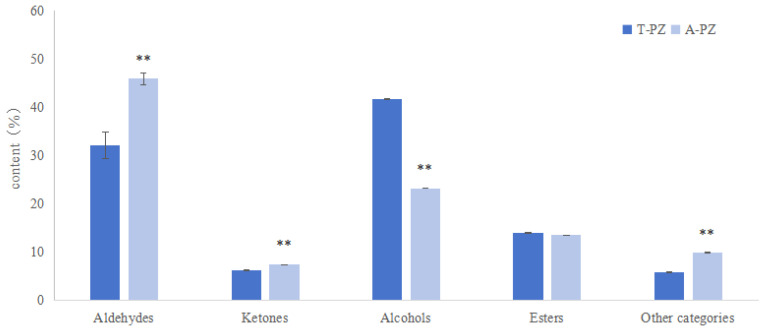
Content of volatile flavor compounds in the flesh of the T-PZ and A-PZ groups (*n* = 3). ** indicates significant difference (*p* < 0.05).

In Figure 3, aldehydes were identified as the predominant volatile compounds in the flesh of both groups, accounting for 32.12% and 45.89% of the total volatile content, respectively, followed by ketones, which accounted for 41.79% and 23.28%, respectively. Compared with the T-PZ group, the proportions of flesh aldehydes, ketones, and other flavor substances in A-PZ were significantly higher (*p* < 0.01), while the proportion of alcohol flavor substances was significantly lower (*p* < 0.01), and there was no significant difference in the proportion of esters between the two groups (*p* > 0.05).

Since the concentration of volatile compounds does not directly correspond to their contribution to overall flavor, sensory characteristics are determined by both compound concentration and odor threshold values [19]. Only volatile compounds with substantial contributions were identified as key flavor components. Using the relative odor activity value (ROAV) method, the contributions of individual volatile components to the overall flavor of Pengze crucian carp were further analyzed, and the odor characteristics of important flavor compounds were described. The results showed that 1-octene-3-ol had both a relatively high content and a low odor threshold in both groups, indicating that it contributed most strongly to overall odor perception. Accordingly, the ROAV_max_ of 1-octen-3-ol was set to 100 to calculate ROAVs of other volatile compounds. Compounds with ROAV ≥ 1 were considered key odorants contributing significantly to the overall aroma, while compounds with 0.1 ≤ ROAV < 1 were regarded as odor-modifying substances [20].

From Table 9, the key flavor components in the muscle of both groups were largely similar. However, the ROAVs of (E)-2-octenal, (E)-2-decenal, and (E,E)-2,4-deceneal in T-PZ were higher than those in A-PZ, while the ROAVs of hexanal, heptanal and nonanal were higher in the A-PZ group. These results indicate that although the key flavor substances were generally similar between the two groups, noticeable differences in overall flavor characteristics still exist.

### 3.7. Metabolomics Analysis

**Figure 4:**
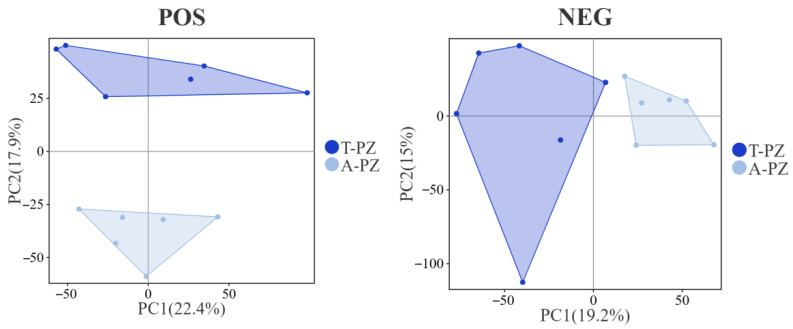
PCA score plots of flesh metabolites in the T-PZ and A-PZ groups. The *x*-axis represents the first principal component (PC1), and the *y*-axis represents the second principal component (PC2). Each point represents an individual sample, and colors indicate different groups.

Principal component analysis (PCA) of muscle metabolites from T-PZ and A-PZ was performed using chromatographic–mass spectrometric techniques (Figure 4). The results showed good intra-group clustering and clear inter-group separation between the T-PZ and A-PZ samples. In the positive and negative ion modes, the contribution rates of PC1 were 22.4% and 19.2%, respectively, while those of PC2 were 17.9% and 15.0%, respectively. Both groups were distributed within the 95% confidence interval. Greater inter-sample variability was observed in T-PZ, whereas higher homogeneity was observed among A-PZ samples. Overall, muscle metabolites could be clearly distinguished between the T-PZ and A-PZ groups in both ionization modes, with more pronounced separation in the positive ion mode.

**Figure 5:**
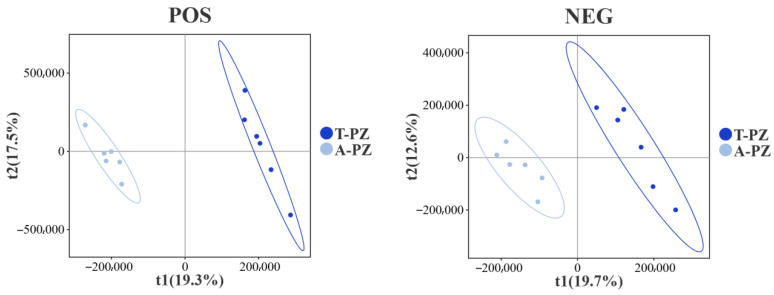
PLS-DA score plots of flesh metabolites in the T-PZ and A-PZ groups. The *x*-axis represents the predictive component (t1), and the *y*-axis represents the orthogonal component (t2). Each point represents an individual sample, and different colors indicate different groups.

**Figure 6:**
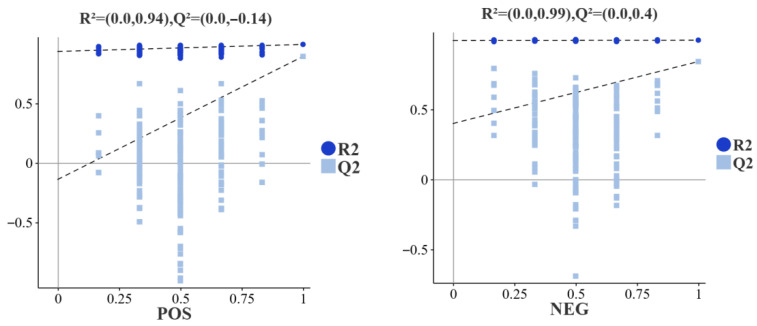
Results of PLS-DA model permutation testing for the T-PZ and A-PZ group. R^2^ and Q^2^ represent the explanatory power and predictive ability of the model, respectively. The *x*-axis indicates the Spearman correlation coefficient between the original and permuted class labels. An R^2^ closer to 1 indicates a better model fit, while Q^2^ reflects model predictability; generally, Q^2^ ≥ 0.4 is considered indicative of a reliable model.

Subsequently, partial least squares method discriminant analysis (PLS-DA) was performed. The score plots (Figure 5) showed clear separation of muscle metabolite profiles between the T-PZ and A-PZ groups in both ionization modes, indicating significant metabolic differences. In the positive ion mode, the model yielded R^2^X = 0.368, R^2^Y = 0.996, and Q^2^ = 0.892, while in the negative ion mode, the model produced R^2^X = 0.446, R^2^Y = 0.995, and Q^2^ = 0.817. According to the permutation test result (Figure 6), the R^2^ and Q^2^ values decreased progressively, and the regression line showed an upward trend, indicating that the permutation test was valid and that the model exhibited good repeatability and predictive ability without overfitting.

**Figure 7:**
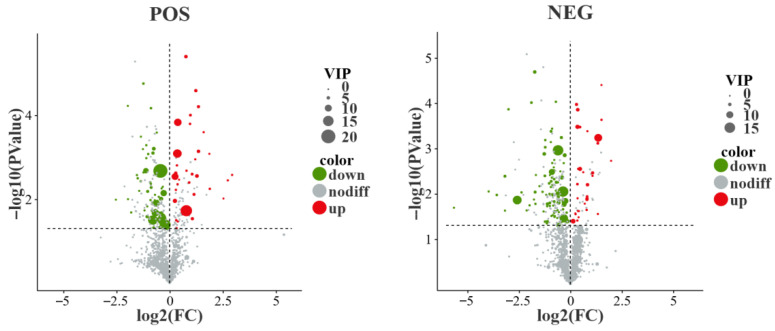
Volcano plots of differential metabolites between the T-PZ and A-PZ groups.

A total of 2380 metabolites were identified in the muscle samples of T-PZ and A-PZ, of which 1306 were detected in the positive ion mode, and 1074 were identified in the negative ion mode. Metabolites satisfying the criteria of VIP ≥ 1 and T-test *p* < 0.05 in the OPLS-DA model were selected as significantly differential metabolites (Figure 7). In the comparative muscle metabolomic analysis of the T-PZ group versus the A-PZ group, a total of 216 significantly differential metabolites were identified, including 93 in the positive ion mode (POS) (36 upregulated and 57 downregulated) and 123 in the negative ion mode (31 upregulated and 92 downregulated). Based on compound classification, the differential metabolites were mainly enriched in carboxylic acids and derivatives (e.g., histidine, L-leucine, L-proline, arginine, D-glutamine, sarcosine, L-threonine, 5-aminolevulinic acid, creatine, betaine, and 1-methyl-L-histidine), fatty acyls (e.g., acetyl-L-carnitine, oleamide, isobutyryl-L-carnitine, D-panthenol, linoleic acid, stearic acid, and oleic acid), and glycerophospholipids (e.g., α-glycerylphosphorylcholine, β-glycerophosphate pentahydrate, 1,2-dioleoyl-sn-glycero-3-phosphocholine, 1-palmitoyl-2-docosahexaenoyl-sn-glycero-3-phosphocholine, and 1-oleoyl-sn-glycero-3-phosphocholine).

**Figure 8:**
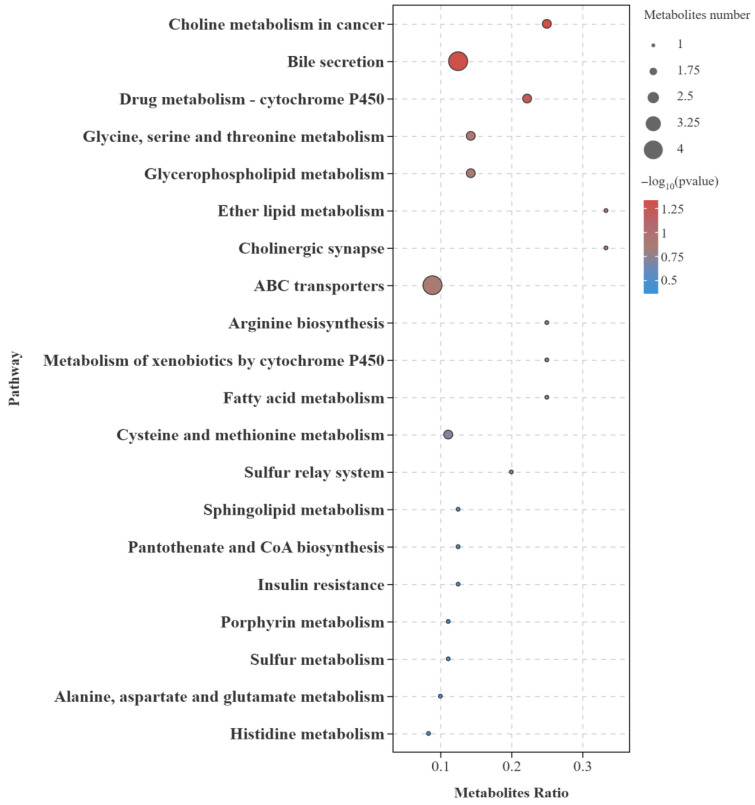
Bubble plot of KEGG metabolic pathway enrichment analysis of significantly differential metabolites in the flesh of the T-PZ and A-PZ groups (top 20). Each bubble represents a metabolic pathway. The abscissa indicates the enrichment ratio, and the ordinate presents the enriched pathways. Bubble size reflects the number of metabolites associated with each pathway. Color corresponds to the *p*-value, with red indicating smaller *p*-values and blue indicating larger *p*-values.

KEGG pathway enrichment analysis was conducted for the significantly differential metabolites. These metabolites were annotated to several secondary pathways, including “Membrane Transport”, “Digestive System”, “Cancers: Overview”, “Amino Acid Metabolism”, and “Metabolism of Cofactors and Vitamins”. Among these, the “Amino Acid Metabolism” pathway was the most highly enriched category, primarily involving six pathways: “Glycine, Serine and Threonine Metabolism”, “Arginine Biosynthesis”, “Cysteine and Methionine Metabolism”, “Alanine, Aspartate and Glutamic acid Metabolism”, “Histidine Metabolism”, and “Biosynthesis of Amino Acids”. In addition, pathways such as “Choline Metabolism in Cancer”, “Bile Secretion”, “Glycerophospholipid Metabolism”, “ABC Transporters”, “Biosynthesis of Secondary Metabolites”, and “Biosynthesis of Unsaturated Fatty Acids” were also significantly enriched. A bubble chart was generated to illustrate the top 20 metabolic pathways with the highest enrichment of significantly differential metabolites (Figure 8).

## 4. Discussion

### 4.1. Muscle Protein and Amino Acid Composition

The nutritional composition of fish flesh, which primarily includes moisture, carbohydrates, mineral elements, crude protein, and crude lipid, fundamentally determines its quality [21]. Protein constitutes a major portion of the dry weight of fish muscle (approximately 78%), while lipids are present in relatively lower amounts and are mainly distributed in the endomysium and perimysium [22]. The abundance of proteins and amino acids is a critical indicator for evaluating muscle nutritional value. In this study, the crude protein content in the muscle of A-PZ was 19.20%, which was significantly lower than that of T-PZ. Nevertheless, this value remains higher than those reported for *C. auratus gibelio* Bloch (17.26%) [23], *C. auratus* Linnaeus (16.69%) [24], and *C. auratus* var. *dongtingking* (18.63%) [25], indicating the high edible value of A-PZ compared to other crucian carp varieties. Li et al. [26] cultivated a new tetraploid gynogenetic crucian carp strain (Changfeng crucian carp) and found that, in contrast to our results, its crude protein content was higher and the crude lipid content was lower than those of a triploid silver carp, suggesting that specific hybrid selection and breeding strategies can significantly influence the basic muscle composition of fish.

Apart from a significantly reduced histidine content in the A-PZ group, no other amino acids showed significant differences between T-PZ and A-PZ. Metabolomic analysis further revealed that histidine and 3-Methylhistidine in A-PZ muscle were significantly downregulated and enriched in pathways such as “amino acid metabolism,” “histidine metabolism,” “amino acid biosynthesis,” and “ABC transporters.” 3-Methylhistidine is a methylated derivative of histidine, which indirectly reflects histidine content and the degradation rate of myofibrillar contractile proteins [27]. Excessive free histidine in fish muscle can be converted to histamine by histamine decarboxylase, and elevated histamine may promote spoilage or inflammatory responses [28,29]. Therefore, the lower histidine level in A-PZ suggests that it may be more conducive to storage, transportation, and secondary processing than T-PZ.

Amino acid degradation is a key reaction of meat aromas [30,31]. The fresh, sweet taste of aquatic products is largely attributed to their free amino acid composition. In this experiment, no significant differences were observed in umami-related amino acids between the two groups, indicating minimal divergence in their foundational flavor profiles. According to FAO/WHO recommendations, a protein is considered high-quality when the ratio of essential to non-essential amino acids (∑EAA/∑NEAA) exceeds 60%, and the ratio of essential to total amino acids (∑EAA/∑TAA) is approximately 40% [32,33]. In this study, the ∑EAA/∑NEAA values for T-PZ and A-PZ were 88.76% and 88.22%, respectively, while the ∑EAA/∑TAA values were 47.10% and 46.87%, respectively, both exceeding the FAO/WHO ideal pattern and confirming that Pengze crucian carp muscle is a high-quality protein source.

The AAS, CS, and EAAI are the key metrics for evaluating protein nutritional value. Values closer to 1 for AAS and CS, and a higher EAAI, indicate superior protein quality and more balanced nutrition [34,35]. Methionine, the initiating amino acid in protein synthesis [36], was identified alongside cysteine as the first limiting amino acid in both AAS and CS evaluations. Therefore, future development of specialized feeds for Pengze crucian carp should consider methionine supplementation. Leucine plays an important role in protein metabolism, immune function, and antioxidant capacity in aquatic animals [37], while lysine is crucial for protein synthesis and nutrient absorption in fish, and can supplement lysine-deficient human diets [38,39]. The contents of leucine and lysine in the muscle of both groups were significantly higher than the FAO/WHO reference pattern and the whole egg protein pattern, with EAAI values exceeding 88%, further attesting to the high nutritional value of Pengze crucian carp.

### 4.2. Muscle Lipid and Fatty Acid Composition

Lipid content and fatty acid composition are vital indicators of the nutritional value of fish. In this study, the crude lipid content in A-PZ muscle was significantly higher than that in T-PZ. An appropriate increase in intramuscular lipid content (within about 2%) can markedly improve texture and juiciness, while higher lipid levels generally indicate greater fatty acid content and nutritional value [40]. Volatile compounds derived from unsaturated fatty acid oxidation contribute to the characteristic aroma of fish [41]. Moreover, certain unsaturated fatty acids cannot be synthesized de novo by humans and must be obtained from their diet [42,43]. Notably, eicosapentaenoic acid (EPA) and docosahexaenoic acid (DHA) offer multiple health benefits, including cancer inhibition, reduction in coronary heart disease incidence, regulation of lipid metabolism, and immune enhancement [44,45], making fish an important dietary source for these fatty acids [46].

Consistent with previous findings, Li et al. [26] reported higher levels of highly unsaturated fatty acids in tetraploid gynogenetic silver crucian carp than in its triploid counterpart. Similarly, Wang et al. [47] found that EPA, DHA, and total polyunsaturated fatty acids (PUFAs) were higher in the fifth-generation gynogenetic silver crucian carp (Zhongke No. 5) than those in Pengze crucian carp. In line with these studies, our results showed that the total PUFAs and EPA + DHA contents were significantly higher in A-PZ than in T-PZ, indicating that selective breeding can enhance the content of nutritionally valuable fatty acids in muscle.

Metabolomics analysis revealed that, compared to T-PZ, linolenic acid, (5Z,8Z,11Z,14Z,17Z)-icosapentaenoyl-CoA, sn-glycero-3-phosphocholine, glycerophosphocholine, and phytosphingosine were upregulated in A-PZ muscle. These metabolites were enriched in pathways such as “glycerophospholipid metabolism,” “biosynthesis of unsaturated fatty acids,” and “fatty acid metabolism”. Linolenic acid can be converted into EPA via desaturase and elongase enzymes and further metabolized to DHA through β-oxidation [48]. (5Z,8Z,11Z,14Z,17Z)-icosapentaenoyl-CoA is a key intermetabolite in EPA synthesis and in membrane phospholipid metabolism, contributing to cellular membrane fluidity and signal transduction [49]. In glycerophospholipid metabolism, sn-glycero-3-phosphoethanolamine and glycerophosphocholine are crucial for phospholipid synthesis; their elevated levels may enhance membrane fluidity and elasticity, potentially improving water retention during cooking and resulting in more tender and juicy meat [50,51]. Phytosphingosine, a component of intestinal microvillar membranes [52], can also activate peroxisome proliferator-activated receptors (PPAR) to reduce systemic inflammation [53]. The upregulation of phytosphingosine in A-PZ may partly explain its observed growth advantages.

### 4.3. Muscle Volatile Flavor Compounds

Flavor in fish muscle arises from volatile compounds through enzymatic degradation and oxidation of precursors. These compounds primarily include aldehydes, alcohols, ketones, and esters. Alcohols in fish mainly originate from lipid oxidation and amino acid degradation [54]. While most alcohols have high odor thresholds and contribute minimally to the overall flavor [55], certain unsaturated alcohols possess lower thresholds and play a more significant role [56,57]. 1-Octen-3-ol, an unsaturated alcohol derived from unsaturated fatty acids, imparts a mushroom-like aroma and has been identified as a key flavor component in several aquatic species [58,59,60]. In this experiment, its content was significantly higher in T-PZ than in A-PZ.

Aldehydes, typically oxidation products of lipids, are major contributors to fish flavor due to their low odor thresholds. Hexanal, heptanal, nonanal, and (E,E)-2,4-decadienal are derived from the oxidation of oleic and linoleic acids. At low concentrations, they impart fresh and pleasant aromas, but at higher levels, they produce undesirable fishy or rancid notes [61]. Hexanal is associated with grassy, rancid, and pungent odors [62], heptanal with fatty, dried-fish, and citrus notes, and nonanal with raw-fish and fatty odors [57]. Compared to T-PZ, A-PZ showed higher relative odor activity values (ROAVs) for hexanal, heptanal, nonanal, and decanal, but lower values for (E)-2-octenal, (E)-2-decenal, and (E,E)-2,4-decadienal.

Ketones in aquatic products are formed through thermal degradation, amino acid degradation, lipid oxidation, microbial oxidation, and the Maillard reaction [57]. They typically exhibit fruity or creamy aromas with higher odor thresholds than aldehydes [63] and can help mitigate fishy odors [64]. Three ketones were detected in this study, with A-PZ showing lower contents, and they can modify or enhance the effects of other flavor compounds [65]. Overall, the volatile compound profiles suggest that T-PZ possesses a superior fresh flavor quality compared to A-PZ.

### 4.4. Muscle Texture and Color Parameters

The color of aquatic animals reflects their physiological state and influences consumer preference and market value. Instrumental color measurement reduces subjective error and provides reliable data for quality assessment [66]. While factors such as rearing environment [67,68,69] and dietary supplementation like astaxanthin [70], carotenoids [71], lutein [72], and algae [73,74] can affect muscle color, no significant differences were observed in the L*, a* and b* of the muscle between T-PZ and A-PZ in this study. This indicates that under identical rearing and dietary conditions, ploidy manipulation did not significantly alter the muscle color of Pengze crucian carp.

Texture is another critical factor influencing consumer acceptance. Texture profile analysis measures parameters such as hardness, springiness, chewiness, cohesiveness, gumminess, and resilience by simulating oral processing [70,75]. Consumers generally prefer firm and elastic meat [76,77]. Increased muscle hardness is often associated with improved quality [78,79], while higher intramuscular lipid content enhances tenderness [80,81]. In this experiment, A-PZ exhibited significantly lower hardness, springiness, gumminess, and chewiness than the T-PZ group, indicating greater tenderness. This inverse relationship with lipid content suggests that allotetraploid Pengze crucian carp has more tender muscle, though the firmer texture of triploid muscle may align better with general chewing preferences.

Muscle hardness is negatively correlated with myofiber diameter and positively correlated with myofiber density [78,82]. Interestingly, Li et al. [26] reported higher myofiber density and greater hardness in tetraploid Changfeng crucian carp compared to triploid forms, which contrasts with our findings. This discrepancy underscores the complexity of texture determinants in fish and warrants further investigation into the intrinsic mechanisms influencing muscle structure and texture in different ploidy variants.

## 5. Conclusions

Compared to triploid Pengze crucian carp, the allotetraploid form exhibits the following muscle quality characteristics: slightly lower protein content but a more balanced amino acid profile; significantly higher levels of polyunsaturated fatty acids, including EPA and DHA, offering greater nutritional and health benefits; and superior tenderness. While no significant differences were observed in muscle color, the triploid form demonstrated a more desirable fresh flavor profile. Overall, the allotetraploid Pengze crucian carp shows advantages in amino acid balance, fatty acid nutritional value, and tenderness, indicating strong potential for targeted breeding and aquaculture applications. Future research should explore the molecular mechanisms underlying these differences and validate the findings in diverse production environments.

## Figures and Tables

**Table 1:** Comparison of growth performance of the T-PZ and A-PZ groups (*n* = 3).

Parameters	T-PZ	A-PZ
IBW (g)	80.55 ± 5.08	84.83 ± 4.79
FBW (g)	140.72 ± 7.13	169.91 ± 7.64 *
WG (%)	74.72 ± 2.01	100.27 ± 1.75 *
Survival (%)	85.77 ± 0.88	88.36 ± 0.34 *
FCR	1.58 ± 0.04	1.51 ± 0.03
CF (g/cm^3^)	3.24 ± 0.16	3.36 ± 0.09

Note: * indicates significant difference (*p* < 0.05).

**Table 2:** Comparison of nutrient composition in the flesh sampled from the T-PZ and A-PZ groups (*n* = 3).

Parameters	T-PZ	A-PZ
Moisture	75.67 ± 0.21	75.53 ± 0.40
Crude lipid	2.70 ± 0.03	3.38 ± 0.02 **
Crude protein	19.77 ± 0.06	19.20 ± 0.10 **
Crude ash	1.15 ± 0.01	1.14 ± 0.01

Note: ** indicates extremely significant difference (*p* < 0.01).

**Table 3:** Comparison of amino acid composition in the flesh sampled from the T-PZ and A-PZ groups (*n* = 3).

Parameters	T-PZ	A-PZ
Threonine ^#^	0.84 ± 0.02	0.84 ± 0.01
Valine ^#^	0.88 ± 0.01	0.87 ± 0.01
Methionine ^#^	0.32 ± 0.12	0.34 ± 0.13
Isoleucine ^#^	0.95 ± 0.02	0.94 ± 0.02
Leucine ^#^	1.98 ± 0.03	1.97 ± 0.02
Phenylalanine ^#^	0.90 ± 0.02	0.89 ± 0.01
Histidine ^#^	0.79 ± 0.01	0.70 ± 0.03 **
Lysine ^#^	1.55 ± 0.02	1.53 ± 0.01
Essential amino acids (EAA)	8.21 ± 0.15	8.09 ± 0.16
Aspartic acid *	1.56 ± 0.03	1.55 ± 0.01
Glutamic acid *	2.12 ± 0.03	2.11 ± 0.02
Glycine *	0.82 ± 0.02	0.81 ± 0.01
Alanine *	1.23 ± 0.02	1.22 ± 0.01
Umami-related amino acids (UAA)	5.73 ± 0.09	5.70 ± 0.04
Serine	0.77 ± 0.01	0.76 ± 0.00
Cysteine	0.18 ± 0.02	0.17 ± 0.01
Tyrosine	0.62 ± 0.01	0.62 ± 0.02
Arginine	1.26 ± 0.03	1.28 ± 0.05
Proline	0.67 ± 0.01	0.65 ± 0.01
Total amino acids (TAA)	17.43 ± 0.30	17.26 ± 0.25
∑EAA/∑TAA (%)	47.10 ± 0.57	46.87 ± 0.49
∑EAA/∑NEAA (%)	88.76 ± 0.82	88.22 ± 0.59
∑DAA/∑TAA (%)	32.87 ± 0.23	33.02 ± 0.14

Note: ^#^ indicates essential amino acid; * indicates umami-related amino acid. ** indicates extremely significant difference (*p* < 0.01).

**Table 4:** Comparison of essential amino acid content in the flesh sampled from the T-PZ and A-PZ groups (mg/g N, wet weight).

Essential Amino Acid	T-PZ	A-PZ	FAO/WHO	Whole Egg Protein
Threonine	266	273	250	292
Valine	278	283	310	411
Isoleucine	300	306	250	331
Leucine	626	641	440	534
Lysine	490	498	340	441
Methionine + Cysteine	158	166	220	386
Phenylalanine + Tyrosine	480	492	380	565
Total	2598	2659	2190	2960

**Table 5:** Comparison of the amino acid scores in the flesh sampled from the T-PZ and A-PZ groups.

Essential Amino Acid	T-PZ		A-PZ	
	AAS	CS	AAS	CS
Threonine	1.06	0.91	1.09	0.93
Valine	0.90 **	0.68 **	0.91 **	0.69 **
Isoleucine	1.20	0.91	1.22	0.92
Leucine	1.42	1.17	1.46	1.20
Lysine	1.44	1.11	1.46	1.13
Methionine + Cysteine	0.72 *	0.41 *	0.75 *	0.43 *
Phenylalanine + Tyrosine	1.26	0.85	1.29	0.87
EAAI	82.17		84.28	

Note: * indicates first limiting amino acid; ** indicates second limiting amino acid.

**Table 6:** Comparison of fatty acid composition in the flesh sampled from the T-PZ and A-PZ groups (*n* = 3).

Parameters	T-PZ	A-PZ
C14:0	16.41 ± 0.09	20.43 ± 0.10 **
C15:0	4.60 ± 0.02	4.75 ± 0.08
C16:0	363.31 ± 0.20	438.17 ± 0.23 **
C18:0	81.12 ± 0.11	101.09 ± 1.03 **
∑SFA	465.44 ± 1.22	564.44 ± 1.04 **
C16:1	54.10 ± 1.01	69.08 ± 0.20 **
C18:1n9t	3.97 ± 0.13	4.54 ± 0.09 *
C18:1n9c	779.01 ± 1.21	1010.31 ± 6.22 **
C20:1	26.11 ± 0.04	40.20 ± 0.08 **
∑MUFA	860.19 ± 3.11	1124.13 ± 10.03 **
C18:2n6c	509.62 ± 0.36	661.11 ± 5.78 **
C18:3n6 GLA	13.48 ± 0.09	10.94 ± 0.03 **
C18:3n3 ALA	67.59 ± 1.06	84.11 ± 1.11 **
C20:2	16.64 ± 0.07	22.41 ± 0.02 **
C20:3n6	51.47 ± 1.16	61.73 ± 1.05 **
C20:4n6	70.93 ± 0.17	58.71 ± 1.07 **
C20:5n3 EPA	6.80 ± 0.01	7.94 ± 0.08 **
C22:6n3 DHA	63.34 ± 1.11	65.43 ± 0.18 *
∑PUFA	799.87 ± 10.41	972.25 ± 12.38 **
∑FA	2125.53 ± 10.11	2660.82 ± 20.99 **
n − 3 PUFA	137.75 ± 1.42	157.50 ± 0.78 **
n − 6 PUFA	645.52 ± 2.11	792.48 ± 4.32 **
∑n − 3 PUFA/∑n − 6 PUFA	0.21 ± 0.00	0.20 ± 0.01
EPA + DHA	70.14 ± 1.12	73.37 ± 1.08 **

Note: ∑FA: total fatty acids; ∑SFA: total saturated fatty acids; ∑MUFA: total monounsaturated fatty acids; ∑PUFA: total polyunsaturated fatty acids; ∑n − 3 PUFA/∑n − 6 PUFA: the ratio of n − 3 PUFA to n − 6 PUFA. * indicates significant difference (*p* < 0.05); ** indicates extremely significant difference (*p* < 0.01).

**Table 7:** Comparison of flesh color parameters between the T-PZ and A-PZ groups (*n* = 6).

Parameters	T-PZ	A-PZ
Lightness (L*)	43.11 ± 0.60	45.39 ± 1.99
Redness (a*)	−1.81 ± 0.17	−2.08 ± 0.29
Yellowness (b*)	6.03 ± 0.49	5.07 ± 0.65

**Table 8:** Comparison of volatile flavor compounds in the flesh of the T-PZ and A-PZ groups (μg/kg) (*n* = 3).

Compound	Retention Time (min)	CAS	T-PZ	A-PZ
Hexanal	6.05	66-25-1	4134.87 ± 54.98	1433.85 ± 55.26 **
Heptanal	9.99	111-71-7	225.24 ± 17.45	52.43 ± 0.98 **
Benzaldehyde	12.57	100-52-7	675.51 ± 3.91	244.32 ± 5.45 **
Octanal	14.74	124-13-0	205.67 ± 2.91	50.28 ± 1.83 **
(E)-2-Octenal	17.4	2548-87-0	459.87 ± 4.11	66.50 ± 0.06 **
Nonanal	19.69	124-19-6	1231.31 ± 52.61	280.78 ± 25.69 **
(E)-4-decenal	23.93	65405-70-1	66.72 ± 3.38	12.85 ± 0.69 **
Decanal	24.44	112-31-2	48.41 ± 2.94	12.88 ± 0.85 **
2,5-Dimethylbenzaldehyde	24.72	5779-94-2	92.99 ± 7.03	34.75 ± 0.98 **
(E)-2-decenal	26.63	3913-81-3	141.07 ± 3.47	29.23 ± 0.42 **
E,(E,E)-2,4-decadienal	27.78	25152-84-5	105.07 ± 4.84	18.96 ± 0.87 **
(E,Z)-2,4-decadienal	28.54	25152-83-4	255.30 ± 24.02	26.11 ± 1.84 **
(E)-4-decenal	29.37	65405-70-1	501.37 ± 26.75	43.58 ± 3.15 **
Tetradecanal	31.4	124-25-4	32.16 ± 1.00	14.38 ± 0.60 **
4-Pentylbenzaldehyde	32.84	6853-57-2	18.80 ± 2.01	-
Tridecanal	34.17	10486-19-8	10.18 ± 0.81	2.23 ± 0.13 **
Pentadecanal	39.02	2765-11-9	39.96 ± 8.13	5.37 ± 0.99 *
Hexadecanal	41.22	629-80-1	39.20 ± 22.23	6.92 ± 0.74
Aldehydes			7796.05 ± 793.31	2335.42 ± 82.60 **
2,5-Octanedione	13.91	3214-41-3	1230.85 ± 79.23	306.82 ± 2.57 **
3-Octen-2-one	16.52	1669-44-9	52.29 ± 3.06	30.19 ± 0.92 **
(E,E)-3,5-Octadien-2-one	19.12	30086-02-3	235.32 ± 7.80	39.59 ± 3.26 **
Ketones			1518.47 ± 83.00	376.60 ± 5.27 **
1-Hexanol	8.71	111-27-3	4308.92 ± 56.80	469.74 ± 13.61 **
1-Octen-3-ol	13.69	3391-86-4	2700.15 ± 204.11	573.41 ± 0.89 **
2-ethyl-1-hexanol	16.09	104-76-7	2037.65 ± 87.55	42.83 ± 1.92 **
(E)-2-Octen-1-ol	17.98	18409-17-1	815.85 ± 20.80	93.46 ± 5.25 **
1-Nonanol	22.97	143-08-8	235.51 ± 4.34	-
Cedrol	36.6	77-53-2	30.41 ± 14.05	4.72 ± 0.53
Alcohols			10,128.48 ± 341.05	1184.17 ± 17.69 **
Isopentyl 3-hydroxy-2-methylenebutanoate	19.25	80758-69-6	2475.93 ± 169.39	160.03 ± 5.96 **
2,2,4-Trimethyl-1,3-pentanediol diisobutyrate	29.7	6846-50-0	-	15.19 ± 0.62
Propanoic acid 2-methyl-3-hydroxy-2,2,4-trimethylpentyl ester	30.36	77-68-9	27.02 ± 1.48	23.34 ± 1.33 *
Carbamodithioic acid, diethyl-, methyl ester	30.55	686-07-7	163.91 ± 6.68	177.89 ± 5.07 *
Dimethyl phthalate	32.69	131-11-3	25.07 ± 1.72	18.00 ± 1.09 **
Butanedioic acid bis(2-methylpropyl) ester	33.32	925-06-4	10.91 ± 0.52	-
1,4-Benzenedicarboxylic acid, dimethyl ester	33.89	120-61-6	26.50 ± 2.24	8.84 ± 0.61 **
2,2,4-Trimethyl-1,3-pentanediol diisobutyrate	36.38	6846-50-0	64.22 ± 11.12	23.83 ± 2.41 **
Hexanedioic acid, bis(2-methylpropyl) ester	38.36	141-04-8	20.19 ± 1.98	-
Tetradecanoic acid, ethyl ester	40.74	124-06-1	18.36 ± 5.93	7.46 ± 0.63
1,2-Benzenedicarboxylic acid, bis(2-methylpropyl) ester	42.32	84-69-5	37.93 ± 12.99	5.12 ± 0.66 *
Hexadecanoic acid, methyl ester	43.43	112-39-0	22.71 ± 11.53	18.84 ± 23.81
Dibutyl phthalate	44.19	84-74-2	35.41 ± 4.98	7.93 ± 0.64 **
Ethyl 9-hexadecenoate	44.36	54546-22-4	63.84 ± 32.04	32.65 ± 2.15
Hexadecanoic acid, ethyl ester	44.76	628-97-7	214.19 ± 140.03	73.78 ± 3.62
Linoleic acid ethyl ester	47.86	544-35-4	63.89 ± 39.99	50.15 ± 3.16
Ethyl Oleate	47.96	111-62-6	110.73 ± 58.69	66.27 ± 4.82
Esters			3380.80 ± 395.58	689.34 ± 42.00 **
Cyclohexene, 1-methoxy-	10.38	931-57-7	382.27 ± 24.60	233.89 ± 4.58 **
2-Hexene, 3,5,5-trimethyl-	13.37	26456-76-8	390.58 ± 5.83	108.85 ± 2.08 **
Formamide, N,N-dibutyl-	28.12	761-65-9	53.88 ± 4.75	13.99 ± 0.65 **
2-Pentene, 4-methyl-, (Z)-	28.9	691-38-3	35.89 ± 1.04	2.86 ± 0.29 **
Cyclohexasiloxane, dodecamethyl-	29.08	540-97-6	70.51 ± 3.62	34.52 ± 0.95 **
2-Dodecyne	30.06	629-49-2	255.61 ± 18.59	22.03 ± 1.78 **
Cycloheptasiloxane, tetradecamethyl-	33.97	107-50-6	20.39 ± 2.44	17.98 ± 1.50
Butylated Hydroxytoluene	34.28	128-37-0	-	14.52 ± 0.45
1,4-Benzenediol, 2,5-bis(1,1-dimethylethyl)-	38.56	88-58-4	111.85 ± 142.14	7.73 ± 0.19
Heptadecane	38.69	629-78-7	40.18 ± 4.55	12.03 ± 1.19 **
n-Hexadecanoic acid	44.09	57-10-3	59.58 ± 16.72	34.62 ± 3.12
Other categories			1420.73 ± 158.39	503.03 ± 4.21 **

Note: * indicates significant difference (*p* < 0.05); ** indicates extremely significant difference (*p* < 0.01).

**Table 9:** ROAVs of volatile compounds in the flesh sampled from the T-PZ and A-PZ groups.

Aroma-Active Compounds	Threshold Value (μg/kg)	Odor Description	ROAV	
			T-PZ	A-PZ
Hexanal	4.5	fishy, grassy	10.89	17.78
Heptanal	2.8	fatty, fishy, citrus	1.53	1.68
(E)-2-Octenal	3	fatty, nutty	2.72	1.86
Nonanal	1.1	fatty, grassy, fishy	53.76	58.27
Decanal	2	grassy, citrus	0.65	0.81
(E)-2-decenal	0.3	Fatty, mushroom	83.59	81.57
(E,E)-2,4-decadienal	0.07	fatty	1143.53	971.71
1-Octen-3-ol	1.2	mushroom	100.00	100.00

## Data Availability

Data will be made available upon request.
